# The farnesoid X receptor agonist obeticholic acid upregulates biliary excretion of asymmetric dimethylarginine via MATE-1 during hepatic ischemia/reperfusion injury

**DOI:** 10.1371/journal.pone.0191430

**Published:** 2018-01-18

**Authors:** Andrea Ferrigno, Laura Giuseppina Di Pasqua, Clarissa Berardo, Veronica Siciliano, Vittoria Rizzo, Luciano Adorini, Plinio Richelmi, Mariapia Vairetti

**Affiliations:** 1 Department of Internal Medicine and Therapeutics, University of Pavia, Pavia, Italy; 2 Department of Molecular Medicine, IRCCS San Matteo, University of Pavia, Pavia, Italy; 3 Intercept Pharmaceuticals, San Diego, California, United States of America; Texas A&M University, UNITED STATES

## Abstract

**Background:**

We previously showed that increased asymmetric dimethylarginine (ADMA) biliary excretion occurs during hepatic ischemia/reperfusion (I/R), prompting us to study the effects of the farnesoid X receptor (FXR) agonist obeticholic acid (OCA) on bile, serum and tissue levels of ADMA after I/R.

**Material and methods:**

Male Wistar rats were orally administered 10mg/kg/day of OCA or vehicle for 5 days and were subjected to 60 min partial hepatic ischemia or sham-operated. After a 60 min reperfusion, serum, tissue and bile ADMA levels, liver mRNA and protein expression of ADMA transporters (CAT-1, CAT-2A, CAT-2B, OCT-1, MATE-1), and enzymes involved in ADMA synthesis (protein-arginine-N-methyltransferase-1, PRMT-1) and metabolism (dimethylarginine-dimethylaminohydrolase-1, DDAH-1) were measured.

**Results:**

OCA administration induced a further increase in biliary ADMA levels both in sham and I/R groups, with no significant changes in hepatic ADMA content. A reduction in CAT-1, CAT-2A or CAT-2B transcripts was found in OCA-treated sham-operated rats compared with vehicle. Conversely, OCA administration did not change CAT-1, CAT-2A or CAT-2B expression, already reduced by I/R. However, a marked decrease in OCT-1 and increase in MATE-1 expression was observed. A similar trend occurred with protein expression.

**Conclusion:**

The reduced mRNA expression of hepatic CAT transporters suggests that the increase in serum ADMA levels is probably due to decreased liver uptake of ADMA from the systemic circulation. Conversely, the mechanism involved in further increasing biliary ADMA levels in sham and I/R groups treated with OCA appears to be MATE-1-dependent.

## Introduction

The farnesoid X receptor (FXR), a member of the nuclear receptor (NR) superfamily, is highly expressed in the liver, intestine, kidneys and adrenals, as well as adipose tissue and vascular walls [1][2][3]. Following activation, FXR binds to the FXR response element (FXRE) in the promoter of its target genes either as a heterodimer with retinoid X receptor (RXR) or, less commonly, as a monomer [4]. Four isoforms of murine and human FXR were identified as a result of alternative promoter usage and alternative splicing of the mRNA [5]. Previous results indicate that FXRa1 (+) isoforms are mainly expressed in cells with an active steroid metabolism, such as hepatocytes, while FXRa2 (+) are the predominant transcripts in other cells of the enterohepatic circuit [6].

Bile acids (BAs) are potent signaling molecules which, through activation of FXR, regulate a wide range of target genes that modulate BA homeostasis, lipoprotein and glucose metabolism and inflammatory responses [7][8]. Recently, Vaquero et al, demonstrated that activation of FXR enhances hepatocyte chemoprotection and liver tumor chemoresistance against genotoxic compounds [9].

The bile acid derivative obeticholic acid (OCA) is a potent FXR agonist [10], recently approved in the US and Europe for the treatment of primary biliary cholangitis (PBC) [11][12]. OCA has also shown beneficial effects in the treatment of nonalcoholic steatohepatitis (NASH) [13].

In the liver, FXR activation induces the expression of canalicular bile transporters such as ABC transporter proteins: bile acid export pump (BSEP, ABCB11), multi-resistance-related protein-2 (MRP-2, ABCC2) and phospholipid flippase (MDR2, ABCB4) [14][15][16]. Previous results have shown that FXR activation by GW4064 leads to up-regulation of cationic amino-acid transporter (CAT-1) and dimethylarginine dimethylaminohydrolase-1 (DDAH-1) in mouse liver and kidney [17]. The enzyme DDAH-1 metabolizes asymmetric dimethylarginine (ADMA), a potent inhibitor of constitutive and inducible nitric oxide synthase (NOS), to citrulline and dimethylamine [18]. In addition, ADMA is able to interfere with NO synthesis by competing with arginine and symmetric dimethylarginine (SDMA), for cellular transport across cationic amino-acid transporters (CATs) [19], belonging to the solute carrier family-7 (SLC7A1-4) [20]. High plasma and liver ADMA levels are found in vascular endothelial DDAH-1-deficient mice and are associated with increased blood [21] and portal [22] pressure, correlated with increased hepatic ADMA levels in patients with alcoholic cirrhosis and superimposed inflammation [22].

The liver abundantly expresses CATs, especially CAT-2A and CAT-2B, and the extensive hepatic expression of CAT-2A mRNA suggests higher ADMA uptake in this organ as compared to the heart, lungs and kidneys [23]. The elimination of ADMA by kidney occurs not just through CAT-2A and CAT-2B but also through the organic cation transporter-2 (OCT-2) while a multidrug and toxin extrusion protein-1 (MATE-1) contributes to its efflux [24]. MATE transporters are members of the solute carrier family-47 (SLC47A1-3), functioning, in particular, as efflux proteins. In 2005, two human MATE transporter proteins, MATE-1 and MATE-2, were identified on the basis of gene sequence similarity [25]. In contrast to other canalicular drug efflux transporters, MATE proteins belong to the SLC47 family [26]. MATE-1 effluxes organic cations using a proton-coupled electroneutral exchange [27]. The organic cation transporters OCT-1, 2, and 3 belong to the solute carrier family-22 (SLC22A1-3) and mediate the facilitated transport of a variety of structurally diverse organic cations, including many drugs, toxins, and endogenous compounds. In the liver, OCT-1 was detected in sinusoidal membranes of hepatocytes around the central veins of the hepatic lobuli [28]. Recently, a down-regulation of OCT-1 gene expression by nuclear receptors, such as FXR and pregnane X receptor, has been reported [29].

We have previously demonstrated that after hepatic I/R damage, biliary ADMA clearance increases, which is associated with changes in CAT-2A and CAT-2B transporters [30][31]. In the present study, we evaluated the effects of the FXR agonist OCA on biliary, serum and hepatic levels of ADMA. In addition, hepatic mRNA and protein expression of ADMA transporters (CAT-1, CAT-2A, CAT-2B, OCT-1 and MATE-1) and the enzymes involved in ADMA synthesis and metabolism, protein methyltransferases-1 (PRMT-1) and dimethylarginine-dimethylaminohydrolase-1 (DDAH-1), respectively, were evaluated.

## Material and methods

### Materials

All reagents were of the highest grade of purity available and were obtained from SIGMA (Italy). The FXR agonist, OCA, was kindly provided by Intercept Pharmaceuticals, San Diego, California, USA.

### Animals and experimental design

Male Wistar rats (Harlan-Nossan, Italy) were used in this study. The animals were allowed free access to water and food in all the experiments. The use and care of animals in this experimental study was approved by the Italian Ministry of Health and by the University of Pavia Commission for Animal Care (Document number 2–2010). Animals were orally administered 10 mg/kg/day of the OCA in methylcellulose 1% vehicle for 5 days or with vehicle alone. After 5 days, blood samples were collected and immediately centrifuged to isolate serum. Hepatic biopsies from the left lobe were collected and snap frozen in liquid nitrogen. The effects of I/R were studied *in vivo* in a partial normothermic hepatic I/R model (n = 8). The rats were anesthetized with sodium pentobarbital (40 mg/kg i.p.), the abdomen was opened via a midline incision and the bile duct was cannulated (PE-50). Ischemia to the left and the median lobe was induced for 60 min with microvascular clips by clamping the branch of portal vein and the branch of the hepatic artery after the bifurcation to the right lobe, with the abdomen temporarily closed with a suture [32]. After 60 min of ischemia, the abdomen was reopened, the clips were removed, the abdomen was closed again, and the liver was allowed to reperfuse for 60 and 120 min. By using partial, rather than total, hepatic ischemia, portal vein congestion and subsequent bacterial translocation into the portal venous blood was avoided. Sham animals were subjected to the same procedure without clamping the vessels (n = 7). To prevent postsurgical dehydration and hypotension, 1 ml of saline was injected into the inferior vena cava. All the animals were maintained on a warm support to prevent heat loss: rectal temperature was maintained at 37±0.1°C. Animals were sacrificed under general anesthesia by exsanguination.

### Serum, bile and tissue sampling

Blood was drawn from the vena cava, immediately placed on ice and centrifuged at 3000 g for 10 min at 4°C. Bile samples were collected into darkened tubes. Hepatic biopsies were quickly removed from the median lobe and immediately frozen in liquid nitrogen, as were bile and serum samples.

### Assays

Liver injury was assessed by serum levels of alanine transaminase (ALT), aspartate transaminase (AST), alkaline phosphatase (ALP), total and direct bilirubin using an automated Hitachi 747 analyser (Roche/Hitachi, Indianapolis, IN, USA).

ADMA levels in plasma, tissue and bile were evaluated by direct ELISA kit according to the manufacturing procedure (Immundiagnostik AG-Germany) [31].

DDAH activity was evaluated using the method proposed by Tain Y-L and Baylis C. [33]. Tissue samples were homogenized in cold phosphate buffer 100 mM, pH 6.5; urease (100 U/ml) was added and samples were incubated at 37°C for 15 min; ADMA 1 mM in a phosphate buffer was added (final ADMA concentration: 0.8 mM) and samples were incubated at 37°C for 60 min; the reaction was stopped by mixing 1:1 with 4% sulphosalicylic acid and samples were centrifuged for 10 min at 3000 g. Finally, the supernatants were assayed for citrulline as follows: Solution A (diacetylmonoxime 80 mM, tiosemicarbazide 2 mM) and Solution B (H_2_PO_4_ 3 M, H_2_SO_4_ 6 M, NH_4_Fe (SO_4_)_2_ 1.75 mM) were prepared, mixed 1:3 and added 1:1 to the samples. Samples were incubated at 60°C for 110 min and read spectrophotometrically at 528 nm against citrulline standards.

### Quantitative real-time PCR analysis of liver

CAT-1, CAT-2A, CAT-2B, OCT-1, MATE-1, DDAH-1, PRMT-1, FXR, HIF-1, mRNA was analyzed by a real-time polymerase chain reaction (RT-PCR): total RNA was isolated from the liver samples with Trizol reagent according to the method of Chomczynski and Mackey [34]. RNA was quantified by measuring the absorbance at 260/280 nm. cDNA was generated using the iScript cDNA Synthesis kit (BIO-RAD) according to the supplier's instructions. Gene expression was analyzed using the SSO Advanced Sybr Green Supermix (BIO-RAD). As regards housekeeping, gene Ubiquitin C (UBC), Tubulin (Tub) and Ribosomal Protein S9 (RS9) were used (Table 1). CAT-1, CAT-2A, CAT-2B, OCT-1, MATE-1, DDAH-1, PRMT-1, UBC, Tub and RS9 gene amplification efficiency was 92.8%, 93.5%, 98.6%, 92.8%, 93.9%, 94.6%, 96.1% and 97.4%, respectively in a cDNA concentration range of 10–0.1 ng/μl. The expression of the housekeeping genes remained constant in all the experimental groups considered. Amplification was performed through two-step cycling (95–60°C) for 40 cycles in a CFX Connect RT-PCR Detection System (BIO-RAD) following the supplier’s instructions. All samples were assayed in triplicate. Gene expression was calculated using the ΔCt method. Comparison between groups was calculated using the ΔΔCt method.

**Table 1:** List of forward and reverse primers used in the experiments.

Gene	Sequence
Rat CAT-1	Forward 5’-GGG TCC GGT TCG CAG TGT GG-3’
	Reverse 3’-GCA CCC GTC AAC CGC TGT CA-5’
Rat CAT-2A	Reverse 3’-GCA CCC GTC AAC CGC TGT CA-5’
	Reverse 3’-TCG TGG CAG CAA CGG GTG AC-5’
Rat CAT-2B	Forward 5’-TAC GTT GTC GCC GCA GGC TC-3’
	Reverse 3’-GCT GCC ACT GCA CCC GAT GA-5'
Rat DDAH-1	Forward 5’-CAA CGA GGT CCT GAG ATC TTG GC-3’
	Reverse 3’-GGA TCA GTA GAT GGT CCT TGA GC-5'
Rat OCT-1	Forward 5’-TCT GTG TCC GGT GTG CTA AC-3’
	Reverse 3’-TGC AGC TCA TGC GGG ATA AA-5’
Rat MATE-1	Forward 5’-TCC CCA TTT ACG CTG TGT CC-3’
	Reverse 3’-ACC ACA GAC CAA TCA CTC CC-5’
Rat PRMT-1	Forward 5’-TGC TGC ACG CTC GTG ACA AGT-3’
	Reverse 3’-TCC ACC ACG TCC ACC AGG GG-5’
Rat UBC	Forward 5’-CAC CAA GAA GGT CAA ACA GGA A-3’
	Reverse 3’-AAG ACA CCT CCC CAT CAA ACC-5’
Rat Tub	Forward 5’-AGA AGC AAC ACC TCC TCC TCG-3’
	Reverse 3’-ATA CAC TCA CGC ATG GTT GCT G-5’
Rat RS9	Forward 5’-CCC TTC GAG AAA TCG CGT CT-3’
	Reverse 3’-GCA GAG CGT TGC CTT CAA AC-5’

CAT-1, cationic amino-acid transporter-1; CAT-2A, cationic amino-acid transporter-2A; CAT-2B, cationic amino-acid transporter-2B; DDAH-1, dimethylarginine dimethylaminohydrolase-1, MATE-1, multidrug and toxin extrusion protein-1; OCT-1, organic cation transporter-1; PRMT-1, protein methyltransferases-1; RS9, ribosomal protein S9; Tub, tubulin; UBC, ubiquitin C.

### Western Blot assay

CelLytic Buffer and Protease Inhibitor Cocktail were purchased from Sigma-Aldrich (Milan, Italy), as well as the mAb anti-alpha-tubulin (DM1A). Rabbit polyclonal antibodies anti-CAT-1 and anti-OCT-1 were from Proteintech (Rosemont, USA), whereas rabbit polyclonal antibodies against CAT-2 and MATE-1 were from Aviva System Biology (San Diego, USA). Rabbit polyclonal antibody against eNOS was purchased from Santa Cruz Biotechnology, Inc., (Dallas, Texas, USA), whereas rabbit polyclonal antibody directed against iNOS was from Cayman Chemical (Ann Arbor, Michigan, USA). Liver tissue samples were homogenized in ice-cold CelLytic Buffer supplemented with Protease Inhibitor Cocktail and centrifuged at 15000 g for 10 min. The collected supernatant was divided into aliquots containing the same amount of proteins, and stocked at -80°C. Samples of liver extracts containing the same amount of proteins were separated in SDS-PAGE on 7.5% or 12% acrylamide gels, and transferred to PVDF membrane. Unspecific sites were blocked for 2 hours with 5% Bovine Serum Albumin (BSA) in TBS (20 mM Tris/HCl, 500 mM NaCl, pH 7.5, 0,1% Tween 20) at 4°C. The membranes were incubated with primary antibodies overnight at 4°C, under gentle agitation. Primary antibodies against alpha-tubulin, CAT-1, CAT-2, iNOS, eNOS were used at a dilution of 1:1000, primary antibodies directed against MATE-1 and OCT-1 were used at a dilution of 1:5000. Membranes were washed in PBS (Na_2_HPO_4_ 8 mM, NaH_2_PO_4_-H_2_O 2 mM, NaCl 140 mM, pH 7.4, 0.1% Tween 20), and incubated with peroxidase-conjugated secondary antibody, at a 1:2000 dilution. Immunostaining was revealed with BIO-RAD Chemidoc XRS^+^. Bands intensity quantification was performed by BIO-RAD Image Lab software.

### Liver histology

At the end normothermic reperfusion, liver biopsies were rapidly removed, fixed in 2% p-formaldehyde in 0.1 M phosphate buffer at pH 7.4 for 24 h and processed routinely until they were embedded in Paraplast wax. Sections were cut at 7 μm and stained with Hematoxylin and Eosin (H&E) for histological examination [35]

### Statistical analysis

Power analysis has been used to calculate group size. Sample size was the average of the values obtained by imposing an effect size ranging from 0.7 and 0.9, a power of 90% and significance of 0.05. Calculations were performed using R Statistical Software (PWR package).

Results are expressed as mean ± standard error (SE). Statistical analysis was performed by ANOVA and Tukey’s test was used for multiple comparisons.

## Results

### FXR agonist OCA induces changes in biliary, serum and tissue ADMA levels

**Fig 1:**
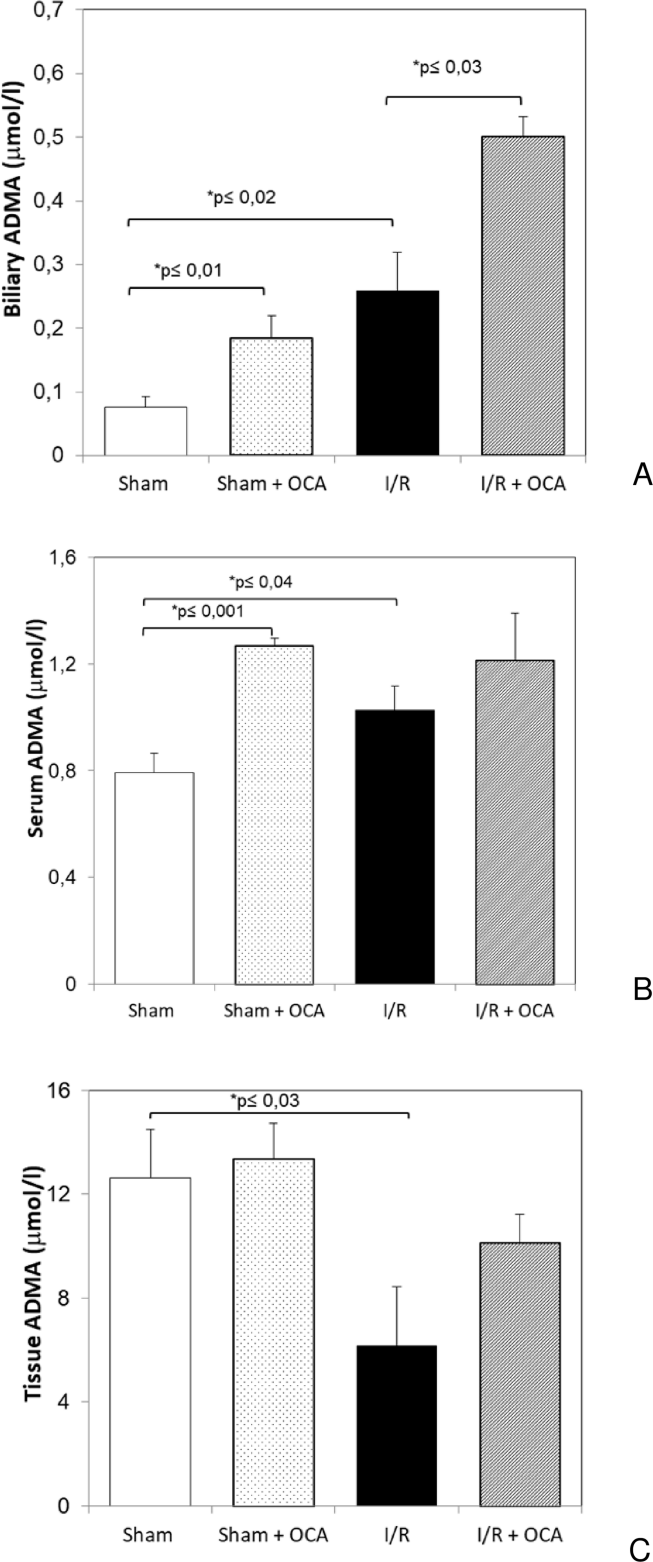
Biliary (Panel A), serum (Panel B) and hepatic (Panel C) changes in ADMA, after I/R in OCA treated rats. Animals were orally administered 10 mg/kg/day of OCA in methylcellulose 1% vehicle for 5 days or with vehicle alone. Livers were submitted to 60 min ischemia followed by 60 min reperfusion. Sham-operated control animals underwent similar manipulation without vascular occlusion. Bile, serum and hepatic samples were collected at the end of reperfusion. The results are reported as the mean ± S.E. of 7–8 different experiments. ADMA, asymmetric dimethylarginine; OCA, obeticholic acid.

OCA administration induced a further increase in biliary ADMA levels both in sham and I/R groups (Fig 1A). A marked increase in serum ADMA levels was found in sham rats treated with OCA compared with vehicle-treated rats: 1.27±0.03 and 0.79±0.07 micromol/l, respectively, p≤0.001 (Fig 1B). No difference in serum ADMA was detected comparing I/R groups with or without OCA treatment. No changes in hepatic ADMA content were found in vehicle and OCA-treated sham groups (pmol/mg prot: 12.6±1.8 and 13.3±1.3, respectively, p≥0.05). An insignificant increase in hepatic ADMA was detected (Fig 1C) in OCA-treated I/R group compared with vehicle-treated I/R rats.

### OCA modulates mRNA and protein expression of ADMA transporters

**Fig 2:**
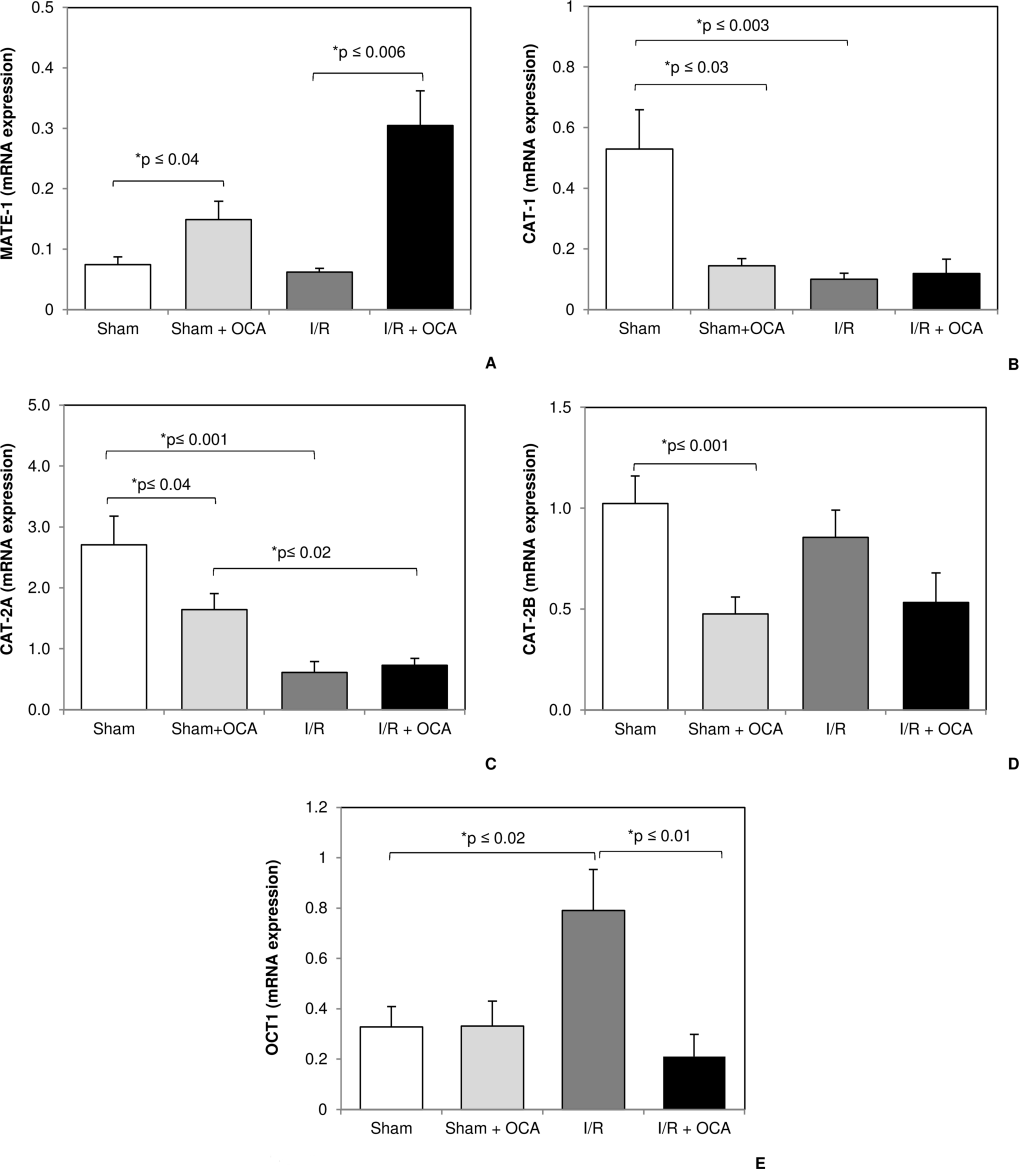
Hepatic mRNA expression of MATE-1 (Panel A), CAT-1 (Panel B), CAT-2A (Panel C), CAT-2B (Panel D) and OCT-1 (Panel E) at the end of reperfusion. Animals were orally administered 10 mg/kg/day of OCA in methylcellulose 1% vehicle for 5 days or with vehicle alone. Livers were submitted to 60 min ischemia followed by 60 min reperfusion. Sham-operated control animals underwent similar manipulation without vascular occlusion. Hepatic samples were collected at the end of reperfusion. The results are reported as the mean ± S.E. of 7–8 different experiments. CAT-1, cationic amino-acid transporter-1; CAT-2A, cationic amino-acid transporter-2A; CAT-2B cationic amino-acid transporter-2B; MATE-1, multidrug and toxin extrusion protein-1; OCA, obeticholic acid, OCT-1, organic cation transporter-1.

Because ADMA was a substrate of MATE-1, CAT-1, CAT-2A, CAT-2B and OCT-1 we evaluated the OCA effects on this transporters in sham and I/R groups. A significant upregulation of MATE-1 was found in both sham and I/R groups treated with OCA compared to vehicle-treated groups (Fig 2A). No increase in MATE-1 occurred in I/R versus sham groups. Conversely, a marked decrease in CAT-1 expression was found in sham rats treated with OCA when compared with vehicle-treated rats (Fig 2B). No difference in CAT-1 was detected comparing I/R groups with or without OCA treatment. A significant reduction in mRNA expression of CAT2A and CAT2B was detected in the sham group after 5 days’ OCA treatment and a decreased CAT-2A expression was found in the OCA group as compared with vehicle-treated rats, 1.64±0.2 vs 2.71±0.4, respectively (p≤0.04) (Fig 2C). The sametrend was detected for CAT-2B expression: 0.47±0.08 vs 1.02±0.1, respectively, p≤0.006 (Fig 2D). A decrease in CAT-2A occurred in I/R groups both in the presence or absence of OCA administration compared to the respective control groups (Fig 2C). Lower CAT-2B expression, although not significant, was observed in the I/R group treated with OCA compared to vehicle-treated I/R rats. OCT-1 showed no changes within the sham groups (Fig 2E). The increase in OCT-1 detected in the I/R group was completely restored to the levels observed in the sham groups by OCA administration (Fig 2E).

**Fig 3:**
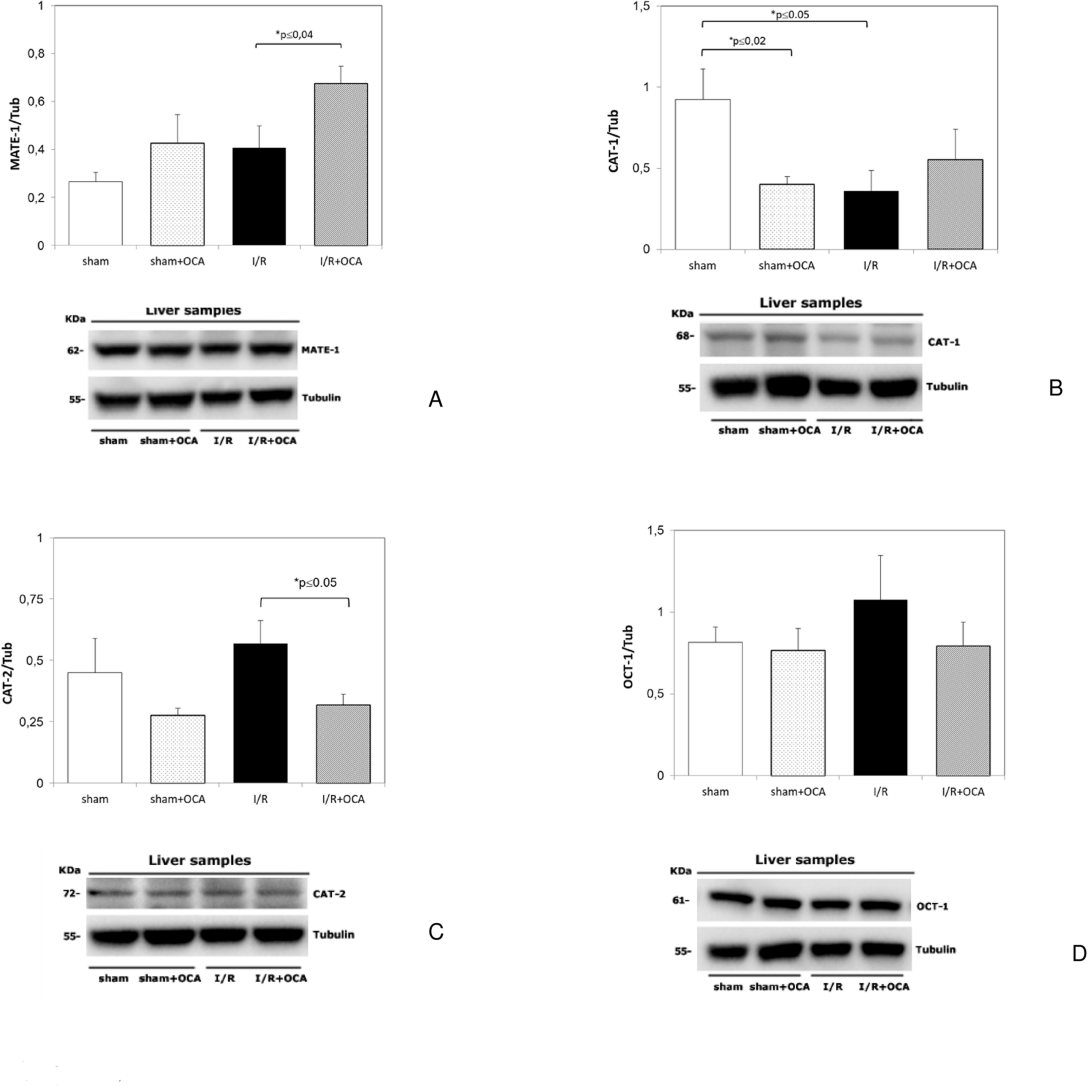
**Hepatic protein expression of MATE-1 (Panel A), CAT-1 (Panel B), CAT-2(Panel C) and OCT-1 (Panel D) at the end of reperfusion.** Animals were orally administered 10 mg/kg/day of OCA in methylcellulose 1% vehicle for 5 days or with vehicle alone. Livers were submitted to 60 min ischemia followed by 60 min reperfusion. Sham-operated control animals underwent similar manipulation without vascular occlusion. At the end of reperfusion, hepatic samples were collected. The results are reported as the mean ± S.E. of 7–8 different experiments. CAT-1, cationic amino-acid transporter-1; CAT-2, cationic amino-acid transporter-2; MATE-1, multidrug and toxin extrusion protein-1; OCA, obeticholic acid, OCT-1, organic cation transporter-1.

The results of the Western Blot analysis demonstrated a comparable trend for ADMA transporters. MATE-1 protein expression was higher in I/R group treated with OCA. An increase in MATE-1 protein, although no significant, was found when comparing sham group with sham + OCA group (Fig 3A). CAT-1 protein showed a comparable trend with the its mRNA expression (Fig 3B). The antibody against CAT-2 is not able to discriminate isoforms CAT-2A and CAT-2B. CAT-2 protein expression was similar to CAT-2B mRNA expression (Fig 3C). Similar trend without any significant difference was found for OCT-1 when comparing I/R group with I/R + OCA group (Fig 3D).

### OCA modulates DDAH activity and mRNA expression

**Fig 4:**
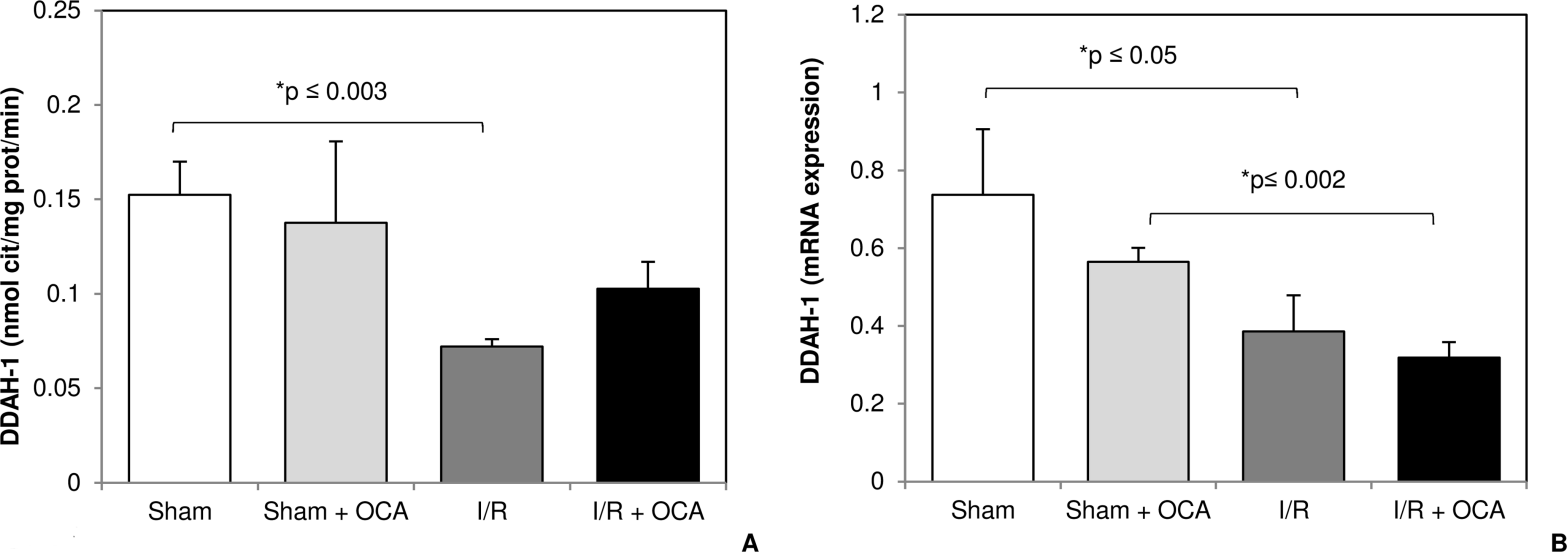
**Hepatic DDAH activity (Panel A) and mRNA expression of DDAH-1 (Panel B) at the end of reperfusion.** Animals were orally administered 10 mg/kg/day of OCA in methylcellulose 1% vehicle for 5 days or with vehicle alone. Livers were submitted to 60 min ischemia followed by 60 min reperfusion. Sham-operated control animals underwent similar manipulation without vascular occlusion. Hepatic samples were collected at the end of reperfusion. The results are reported as the mean ± S.E. of 7–8 different experiments. DDAH-1, dimethylarginine dimethylaminohydrolase-1; OCA, obeticholic acid.

DDAH is the main enzyme involved in ADMA metabolism. In this study we evaluated DDAH mRNA expression and activity finding no detectable changes after 5 days’ OCA administration either in sham or I/R rats (Fig 4). Although not significant, an increase in DDAH activity was found in the I/R group treated with OCA compared with I/R rats (Fig 4A), whereas a significant decrease was found comparing DDAH-1 expression in sham versus I/R groups both in the presence and absence of OCA treatment (Fig 4B).

### OCA reduces hepatic expression of protein methyltransferase (PRMT-1) mRNA and increases eNOS and decreases iNOS protein expression in I/R livers

**Fig 5:**
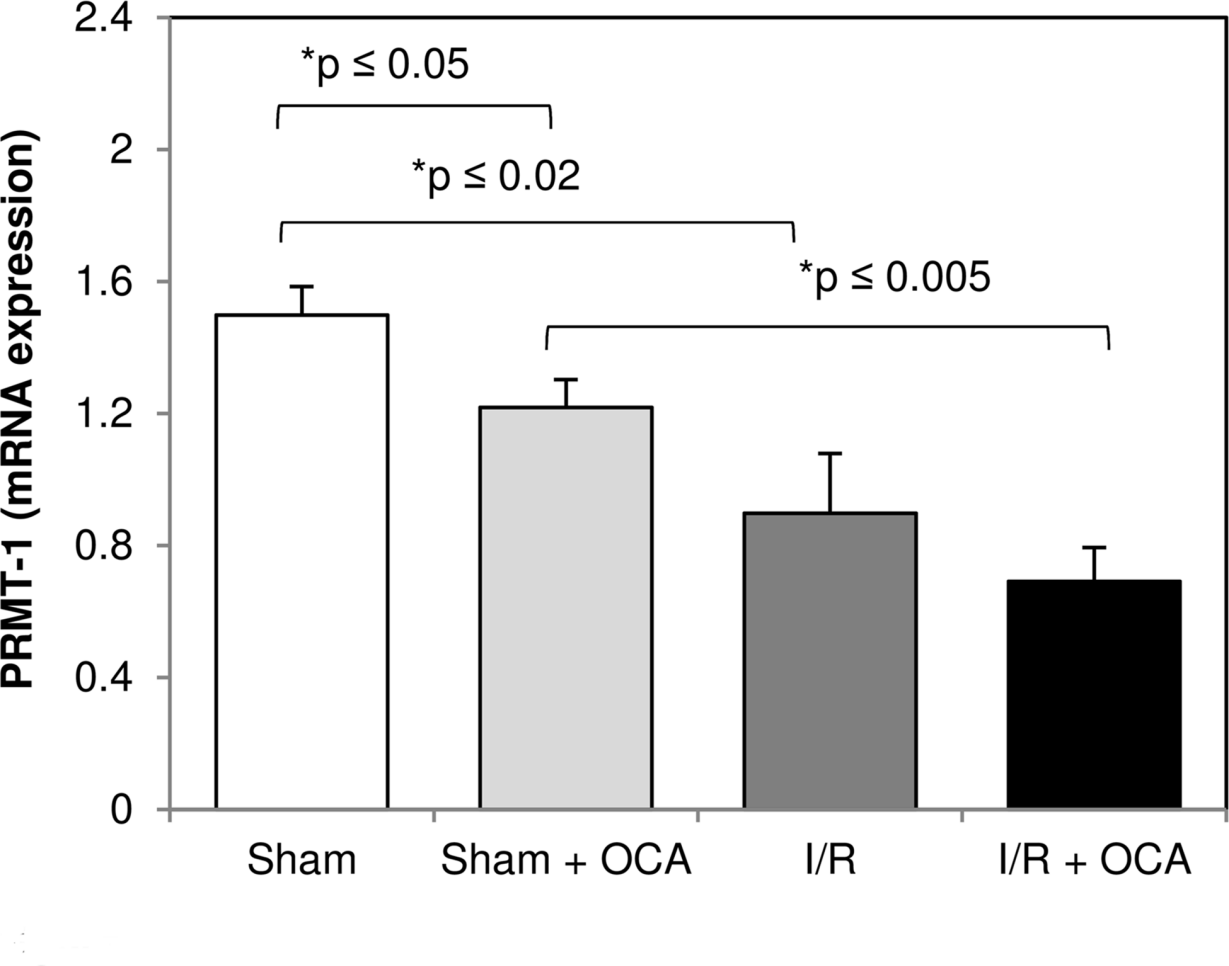
Hepatic mRNA expression of PRMT-1 at the end of reperfusion. Animals were orally administered 10 mg/kg/day of OCA in methylcellulose 1% vehicle for 5 days or with vehicle alone. Livers were submitted to 60 min ischemia followed by 60 min reperfusion. Sham-operated control animals underwent similar manipulation without vascular occlusion. Hepatic samples were collected at the end of reperfusion. The results are reported as the mean ± S.E. of 7–8 different experiments. OCA, obeticholic acid; PRMT-1, protein methyltransferases-1.

In order to assess its significance in ADMA synthesis, PRMT-1 expression was detected after 5 days’ OCA treatment: a significantly lower hepatic expression was found in sham rats treated with OCA as compared with vehicle-treated rats (Fig 5).

**Fig 6:**
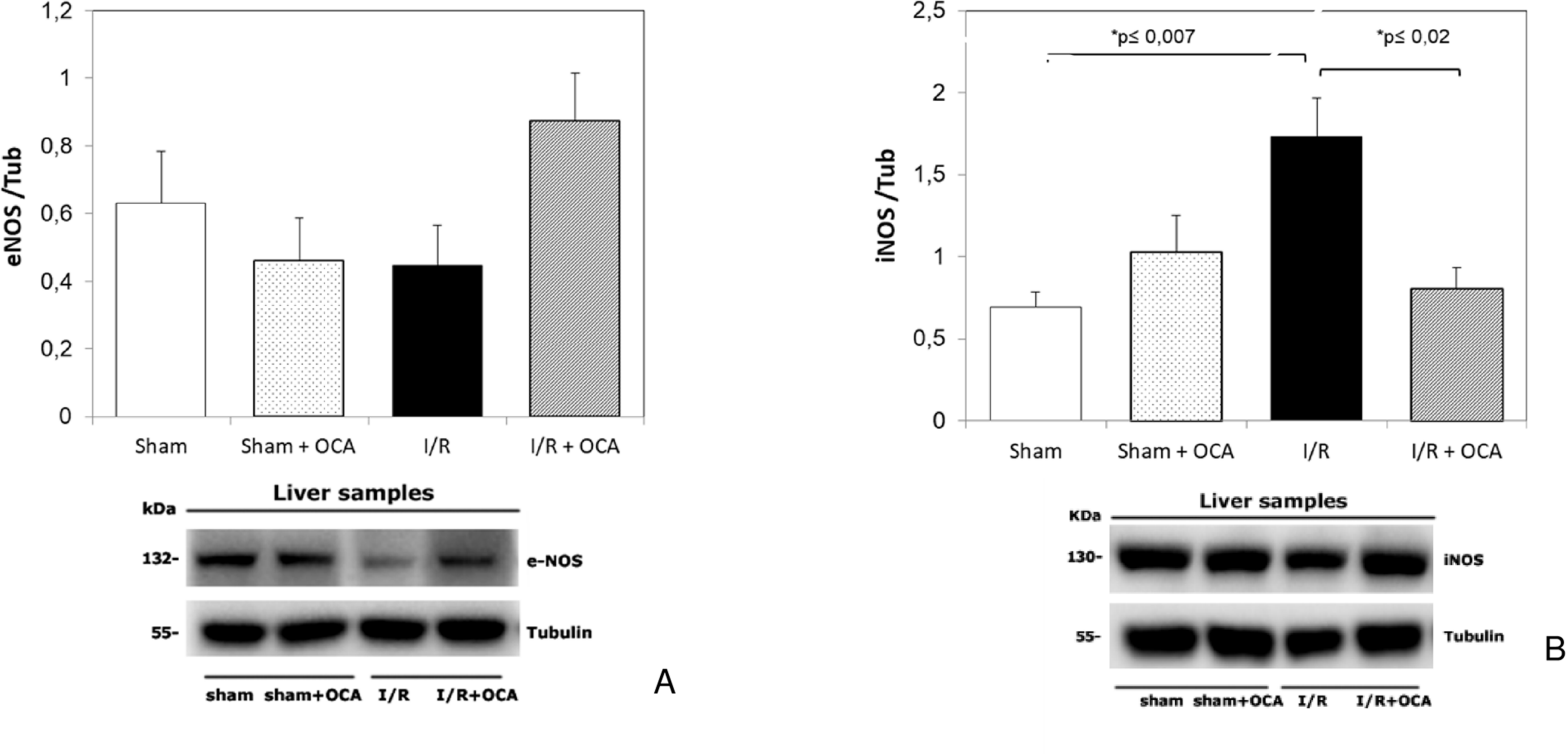
Hepatic iNOS and eNOS at the end of reperfusion. Animals were orally administered 10 mg/kg/day of OCA in methylcellulose 1% vehicle for 5 days or with vehicle alone. Livers were submitted to 60 min ischemia followed by 60 min reperfusion. Sham-operated control animals underwent similar manipulation without vascular occlusion. Hepatic samples were collected at the end of reperfusion. The results are reported as the mean ± S.E. of 7–8 different experiments. OCA, obeticholic acid.

Previous studies demonstrated OCA effects on eNOS and iNOS. In the present study we demonstrated that treatment with OCA induced an increase, although not significant, in eNOS in the I/R group (Fig 6A). However, OCA treatment decreased iNOS expression after I/R as compared with vehicle-treated animals (Fig 6B). No significant changes occurred between sham and OCA-treated sham groups (Fig 6).

### OCA does not change the I/R damage

No changes in serum AST, ALT, AP, total and direct bilirubin levels were observed when comparing OCA-treated sham rats with the vehicle-treated sham group (Table 2). Although not significantly, OCA administration reduced hepatic serum enzyme levels in the I/R group (Table 2). The same trend was found for serum levels of total bilirubin, while a significant decrease was detected for direct bilirubin in the I/R group treated with OCA compared with vehicle-treated I/R rats (Table 2).

**Table 2:** Effects of OCA on serum biochemical parameters in sham and I/R animals.

	Sham	Sham + OCA	I/R 60/60	I/R 60/60 + OCA	I/R 60/120	I/R 60/120 + OCA
AST (mU/ml)	243±57	141±23	3572±906	2457±162	9821±899	8602±382
ALT (mU/ml)	66±17	47±7	3785±802	2733±169	9063±763	8692±343
ALP (mU/ml)	410±46	379±34	582±50	620±62	732±88	704±74
Total Bilirubin (mU/ml)	0.14±0.018	0,10±0.004	0,250±0.05	0,201±0.07	0,299±0.07	0,239±0.07
Direct Bilirubin (mU/ml)	0.045±0.014	0,033±0.002	0,17±0.03	0,082±0.01*	0,19±0.04	0,084±0.03*

*p<0.05 vs I/R. ALT, alanine transaminase; ALP, alkaline phosphatase; AST, aspartate transaminase.

**Fig 7:**
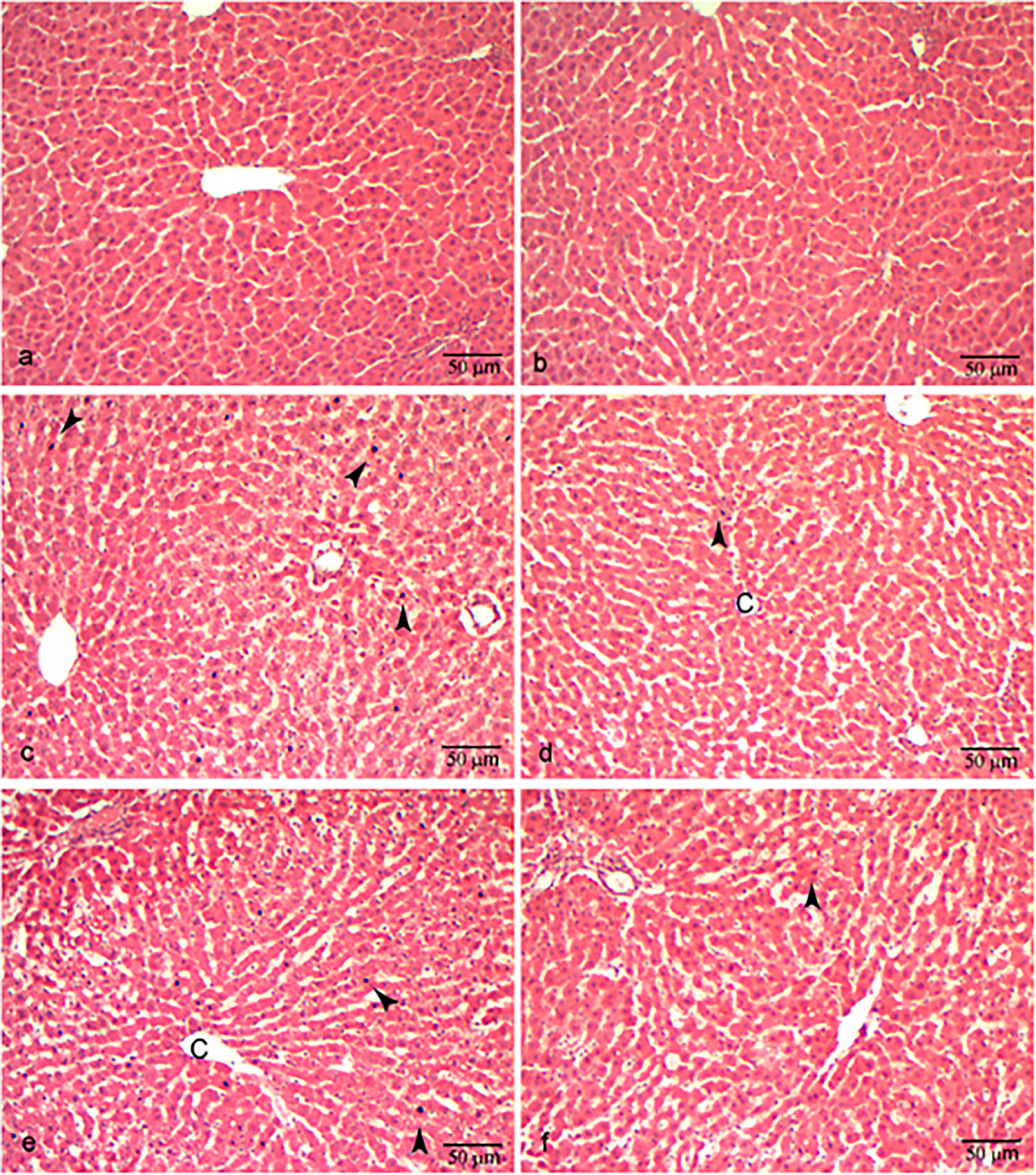
Representative photomicrographs of the morphology (Hematoxylin and eosin staining) of sham-operated and ischemic rat livers. (a) Livers from sham operated rats showed a well-preserved parenchymal architecture and good cellular morphology; (b) livers from sham-operated rats treated with OCA did not differ from control sham rats; (c) livers from rats undergoing 60 min ischemia and 60 min warm reperfusion presented pale-stained vacuolated hepatocytes with pyknotic nuclei, loss of parenchymal architecture and intercellular border; (d) animals treated with OCA and submitted to 60 min ischemia and 60 min reperfusion did not differ significantly from control 60 min ischemia reperfusion livers; (e) livers undergoing 60 min ischemia and 120 min reperfusion showed wider areas of necrosis and loss of intercellular borders and parenchymal disarrangement with respect to 60 min ischemia and 60 min reperfusion livers; (f) rats treated with OCA, when submitted to 60 min ischemia and 120 min reperfusion, did not show significantly different morphologies when compared to non-treated ischemic livers. Arrows: pyknotic nuclei; C: centrilobular vein. Scale bar, 50 μm.

The degree of histological hepatocyte damage strongly differed between sham operated rats and rats undergoing ischemia/reperfusion. Livers from sham-operated animals showed well-preserved hepatic architecture. Ischemia/reperfusion caused marked injury to the parenchyma with sinusoid dilatation, extensive areas of nuclear pyknosis and cytoplasmic vacuolation and wide areas of necrotic cells detached from the parenchyma, especially after 120 min reperfusion. OCA did not change the liver morphology although we found less pyknotic cells in liver specimens from OCA treated rats (11.9±1.2 versus 7.7±0.9 in I/R 60/60 vs I/R 60/60 + OCA, p≤0.01; 13.7±1.3 versus 8.7±1.1 in I/R 60/1200 vs I/R 60/120 + OCA, p≤0.04) (Fig 7).

## Discussion

This work has documented for the first time that OCA, a FXR agonist, further increases excretion of ADMA in bile during hepatic I/R injury. Since ADMA transporters such as CATs and OCT-1 were markedly downregulated and MATE-1 was upregulated in the I/R group treated with OCA, we suggest that MATE-1 proteins, involved in the excretion of compounds into bile, are also responsible for the biliary excretion of ADMA as they provide an alternative elimination route.

MATE efflux transporters are H^+^-coupled antiporters, expressed in the liver and kidneys and localized at apical membranes of renal tubular epithelia and bile canaliculi [36]. MATE-1 (SLC47A1) accounts for excretion of organic cations across the apical membrane in the liver, and we propose its involvement in the further increase in biliary excretion of ADMA observed in I/R rats treated with OCA. In the present study, we also documented a downregulation of CAT-1, CAT-2A, CAT-2B and OCT-1 transporters in the I/R group treated with OCA. OCA was shown to be responsible for the increased ADMA clearance by bile in the sham group as well, representing an FXR-mediated effect associated with a decrease in CAT transporters. The function of MATE-1 is pH dependent [24] and extracellular acidification improves the export function of MATE-1. pH acidification typically occurs in ischemic tissue and provides substantial protection against anoxic damage [37] [38]. The above evidence helps to contextualize the results of the present study: the increased ADMA clearance by bile that takes place in the I/R group occurs through MATE-1 and may represent an adaptive mechanism that limits dangerous hepatic accumulation of ADMA. Because high levels of ADMA appear to be detrimental for mitochondrial function [39], it is reasonable to assume that the liver attempts to protect mitochondria already suffering from ischemia. Previously, elevated endogenous ADMA was found to contribute to hepatic mitochondrial dysfunction in diabetic rats and recent evidence has shown that ADMA can also be imported into mitochondria by SLC25A2 transporters [40].

ADMA, a polar hydrophilic amino acid commonly found in serum, enters into hepatocytes, is imported into mitochondria and is excreted into the bile through cationic transporters.

In the present study, OCA treatment induced an impairment in ADMA exchange by decreasing its transporters, CATs and OCT-1. These might well explain the additional increase in biliary ADMA observed in I/R animals treated with OCA. As we have shown, all CAT isoforms were decreased in the sham group treated with OCA. CAT-2B isoforms are low capacity transporters with a high affinity for cationic amino acids and, in particular, high affinity for ADMA [41]. In contrast, CAT-2A, an alternate splice variant of CAT-2B, possesses low affinity but a high transport capacity. In our study, a decrease in CAT-1 and CAT-2A isoforms was associated with the ischemia injury; OCA administration further reduces CATs, in particular the CAT-2B isoform both in sham and I/R groups. At variance with our data, the synthetic non-steroidal FXR agonist, GW4064, induced an increase in CAT-1 and a decrease in plasma levels of ADMA in mice [17]. A possible explanation for this discrepancy could be related to the high doses of GW4064 used. In addition, GW4064 presents poor bioavailability and limited oral exposure, whereas OCA shows a highly efficient enterohepatic circulation, with pharmacokinetics and metabolism similar to chenodeoxycholic acid (CDCA) [42][43].

The modulation in OCT-1 expression observed in our model of hepatic I/R is noteworthy. This transporter also belongs to the SLC family and its involvement in ADMA efflux/uptake has been recently recognized [24]. In humans, OCT-1 is mainly found in the liver and is located in the hepatocytes and to a lesser extent in cholangiocytes [36]. The present study documents that administration of OCA markedly decreases OCT-1 mRNA expression in the I/R group. Conversely, no changes in the sham group with or without OCA treatment were found. Transport by OCT transporters is classified as facilitated diffusion because OCTs translocate the substrate in both directions across the membrane and function as electrogenic uniporters of organic cations [29]. The activation of FXR by bile acids appears to be associated with the cell-specific expression of membrane transporters [44]. We note that previous data demonstrated that cholic acid treatment led to a significant decrease in OCT-1 expression using wild-type mouse liver [45]. Given that OCTs typically support basolateral organic cations entry and MATEs support apical organic cations efflux [46], it follows that, in the liver, OCT-1 functions in concert with MATE-1 to mediate hepatic uptake and biliary excretion, respectively, of cationic drugs and their metabolites [47]. However, we don’t exclude that the biliary excretion of ADMA could be independent of the changes observed in CATs and OCT-1 basolateral transporters and alternative pathways and transporters may be involved.

Recently, OCA has been found to increase DDAH-1 gene expression in a dose-dependent fashion in HepG2 cells [48]. In addition, animals treated with high-salt diet and OCA at 10 or 30 mg/kg/day for 6 weeks exhibited high DDAH-1 protein [49]. Administration of OCA in a model of cirrhosis induced by bile duct ligation (BDL) induced an increase in DDAH-1 protein [48] and an increase in serum ADMA [11], while in the present study regular biliary clearance allows ADMA to be eliminated and OCA did not alter DDAH-1 expression or activity during I/R. An explanation for our data emerges when the different mechanisms controlling ADMA elimination are compared in the two experimental models, as it is only in the I/R model but not in the BDL model that ADMA can be eliminated by bile. Different ADMA clearance is associated with different hepatic ADMA concentrations, elevated in the BDL model but not in the I/R model, which has effects on DDAH-1 expression.

FXR activation up-regulates eNOS mRNA and protein in vascular endothelial cells [50]. More recently, OCA administration in a cirrhosis model was seen to increase hepatic eNOS activity and this in turn was concomitant with a reduction in portal pressure [48]. The activation of FXR, also by its endogenous ligand CDCA, in hypertensive rats upregulates eNOS expression reducing blood pressure [51]. In addition, in endothelial cells, OCA treatment has been shown to upregulate eNOS expression and enhanced NOS activity induces vasodilation [52]. The present study once more confirms OCA’s ability to increase eNOS levels and demonstrates a decreased iNOS content. Both eNOS and iNOS are involved in the pathogenesis of hepatic I/R injury: eNOS mediates protection whereas iNOS synthetizes NO resulting in hemodynamic instability during hepatic I/R [53][54]. Furthermore, OCA’s ability in decreasing iNOS was recently observed in a rat model of toxic cirrhosis [55].

Recent data have reported the efficacy of OCA in patients with PBC and OCA inducing a significant decrease in alkaline phosphatase and bilirubin, both biochemical markers of PBC outcomes [56][57]. In the present study, using an I/R model, a significant decrease in direct bilirubin was similarly found to be induced by OCA. In a rat model of toxic cirrhosis, OCA was shown to be able to reduce fibrosis both during ongoing cirrhogenesis and also in established cirrhosis [55], suggesting an explanation for the positive results observed in NASH patients [13].

Results obtained in a rat model of intestinal I/R injury have recently demonstrated that pretreatment with OCA improves survival, preserves mucosal integrity, inhibits bacterial translocation and reduces pro-inflammatory cytokine release [58]. Our study provides novel insights into the effects of OCA by showing its capacity to increase MATE-1 expression. FXR plays a central role in BA homeostasis in particular by regulating genes involved in BA secretion and reabsorption [7]. FXR activation by BAs upregulates bile salt export pumps (BSEPs) and multidrug resistance protein 2 (MRP2) mainly responsible for BA transport at the canalicular membrane. These events are associated with an FXR-mediated down-regulation of the Na^+^-dependent taurocholate transporter (NTCP) responsible for basolateral BA transport into the hepatocyte, thus protecting the liver from toxic accumulation of BAs [59]. Here we have reported that once activated by OCA, FXR also upregulates expression of MATE-1, a canalicular drug efflux transporter. Of note, BAs have been found to initiate the expression of transporters in newborn mice via FXR activation and also MATE-1 is reported as increased [60].

## Conclusions

**Fig 8:**
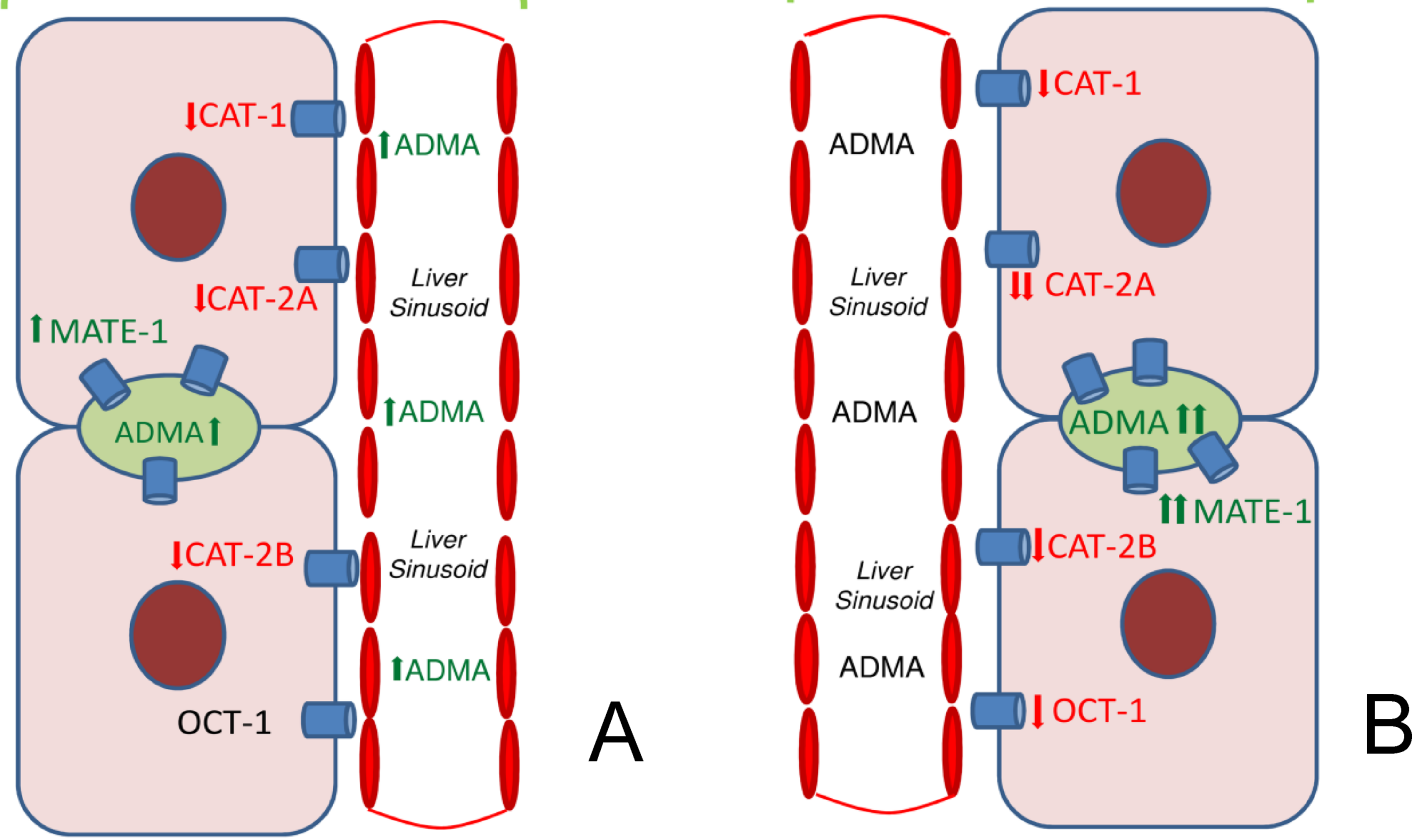
**Schematic representation of changes in CAT-1, CAT-2A, CAT-2B, OCT-1 and MATE-1 transporters that occur in rat livers after OCA treatment (panel A) or hepatic I/R and OCA administration (panel B).** CAT-1, cationic amino-acid transporter-1; CAT-2A, cationic amino-acid transporter-2A; CAT-2B cationic amino-acid transporter-2B; MATE-1, multidrug and toxin extrusion protein-1; OCA, obeticholic acid, OCT-1, organic cation transporter-1.

Our results further support the role of FXR, the major BA sensor, in the control of a wide spectrum of hepatic transporters. In particular, OCA induces biliary excretion of ADMA both in sham and I/R conditions by upregulating MATE-1 (Fig 8). We also demonstrate OCA’s ability to limit the efficiency of the basolateral ADMA transporters in I/R injury, thus favoring further ADMA excretion by bile.
